# Over-The-Scope-Clip pre-mounted onto a double balloon enteroscope for fast and successful closure of post-EMR jejunal perforation: case report

**DOI:** 10.1186/s12876-017-0718-2

**Published:** 2017-12-08

**Authors:** Flaminia Purchiaroni, Takeshi Nakajima, Taku Sakamoto, Seiichiro Abe, Yutaka Saito

**Affiliations:** 1 Wolfson Unit for Endoscopy, St Mark’s Hospital, London, UK; 2 Endoscopy Division, National Cancer Center Hospital, 5-1-1 Tsukiji, Chuo-ku, Tokyo, 104-0045 Japan; 3 Department of Genetic Medicine and Services, National Cancer Center Hospital, Tokyo, Japan

**Keywords:** Familial adenomatous polyposis, Jejunal polyps, Jejunal perforation, Double balloon enteroscopy, Pre-mounted over the scope clip

## Abstract

**Background:**

Familial adenomatous polyposis (FAP) is a rare, autosomal dominant disease clinically characterized by the early onset of many adenomatous polyps throughout the colon, which turn into colon cancer, if left untreated. In FAP patients, polyps can also occur in the upper gastrointestinal (GI) tract, especially in the duodenum. Adenomas beyond duodenum are rare and mostly located in the proximal jejunum and distal ileum. The management of such polyps can be either surgical or endoscopic, depending on the features of the polyp, Spigelman stage and patient’s clinical conditions. Endoscopic mucosal resection (EMR) of jejunal polyps can be challenging, because of the thinner wall of jejunum, compared to the rest of the GI tract, and of the difficulty of maintaining control and stability of the scope. For these reasons, jejunal perforation is a likely occurrence.

**Case presentation:**

A 65-year-old woman with a stage IV FAP, who had previously undergone abdominal surgery because of her disease, came to our attention because of numerous adenomatous-looking duodenal polyps and a 25 mm lesion in proximal jejunum. According to Spigelman staging system, patient was candidate for surgical resection, in light of the risk of developing small bowel cancer. Despite the benefits of surgery were clearly explained to her, she refused to undergo small bowel resection. Therefore, EMR of the largest duodenal polyp and of the jejunal lesion was planned. After the removal of the jejunal polyp, a small perforation was noted. We were able to rapidly close such perforation by using the Over-The-Scope-Clip system (OTSC, 12/6 t; Ovesco, Tübingen, Germany) pre-mounted onto a double balloon (DB) enteroscope.

**Conclusions:**

The endoscopic management of jejunal perforation can be tricky and the placement of traditional through-the-scope clips in a narrow space like jejunum may be difficult and time consuming.

This case describes the use of the OTSC system pre-mounted onto a DB enteroscope for the closure of post-EMR jejunal perforation.

## Background

Familial adenomatous polyposis (FAP) is an autosomal dominant disease caused by mutations in the adenomatous polyposis coli (APC) gene. It is characterized by the early onset of hundreds to thousands of adenomatous polyps throughout the colon, which turn into colon cancer, if left untreated. FAP is also associated to an increased risk of cancer in the stomach and/or small bowel, as well as in other organs (such as liver, thyroid, pancreas, adrenal glands and bones) [1]. The only strategy to decrease mortality from colonic malignancy is colon screening with subsequent prophylactic surgery [2]. FAP patients also need upper gastrointestinal (GI) screening, with standard endoscopy and side-view duodenoscopy, for gastric and duodenal lesions. Adenomas beyond duodenum are rare and most of the times located in the proximal jejunum and distal ileum [3]. Such lesions are usually found in patients with duodenal adenomas and Spigelman stage ≥ II [4] and can be detected using capsule endoscopy (CE) and/or double balloon (DB) enteroscopy [4, 5].

Treatment of small bowel adenomas can be either surgical or endoscopic, depending on Spigelman stage [6] (Table 1). Balloon-assisted endoscopic mucosal resection (EMR) of jejunal polyps can be challenging, because of the thinner wall of jejunum, compared to the rest of the GI tract, and of the difficulty of maintaining control and stability of the enteroscope. For these reasons, perforation is a likely occurrence, which requires a prompt management in order to avoid lethal consequences.

**Table 1:** Treatment of duodenal polyps depending on Spigelman stage

Spigelman stage	Points	Treatment
0-I	0–4	None
II	5–6	Consider ET^a^ + CP^b^
III	7–8	ET^a^ + CP^b^
IV	9–12	Surgery (PPPD^c^)

^a^ET, endoscopic therapy

^b^CP, chemoprevention with non-steroidal anti-inflammatory drugs (NSAIDs)

^c^PPPD, pancreas and pylorus preserving pancreaticoduodenectomy

Here we report the case of a woman with FAP who experienced small bowel perforation after EMR of a jejunal polyp. Such perforation was rapidly treated using a DB enteroscope with a pre-mounted Over-The-Scope-Clip system (OTSC, 12/6 t; Ovesco, Tübingen, Germany), a relatively new tool for the endoscopic entrapment of tissue for the closure of fistulas and perforation.

## Case presentation

A 65-year-old woman with a long history of FAP and no other medical problem came to our attention because of many duodenal polyps found at surveillance esophagogastroduodenoscopy (EGD). The previous EGD was dated about 10 years ago, as she refused to be followed up for many years, and did not show any small bowel abnormalities. In 1970 she underwent colectomy with ileorectal anastomosis (IRA) and, in 2001, she further underwent restorative proctocolectomy with ileal pouch-anal anastomosis (IPAA).

The most recent surveillance EGD showed numerous adenomatous-looking duodenal polyps and the largest one was 40 mm in size. According to Spigelman staging system, she had a stage IV disease and, therefore, she was candidate for duodenal resection, in light of the risk of developing duodenal cancer. Despite the benefits of surgery were clearly explained to the patient, she refused to undergo duodenal resection. Therefore, we performed EMR of the largest polyp and histology revealed tubulovillous adenoma with high-grade dysplasia.

Since it was likely to find polyps beyond duodenum, as the patient had a Spigelman stage IV, she also underwent surveillance CE. The latter showed a 25 mm Paris 0-IIa polyp beyond Treitz ligament, in proximal jejunum. Again, patient was not willing to undergo surgical resection and, therefore, an EMR was planned. We were concerned about the risk of perforation, because jejunal wall is thin, polyp had a flat morphology and patient had adhesions from previous IRA and IPAA, which made the possibility of perforation even higher. Moreover, if jejunal perforation occurs, its endoscopic management may be challenging, as the placement of traditional through-the-scope clips in a narrow space like jejunum is usually difficult. Therefore, we had to carefully think in advance about a fast endoscopic treatment in case of perforation, in order to avoid emergency surgery, which was risky, due to the presence of adhesions from previous IRA and IPAA. We believed that OTSC system was a good option to deal with a possible jejunal perforation.

**Fig. 1:**
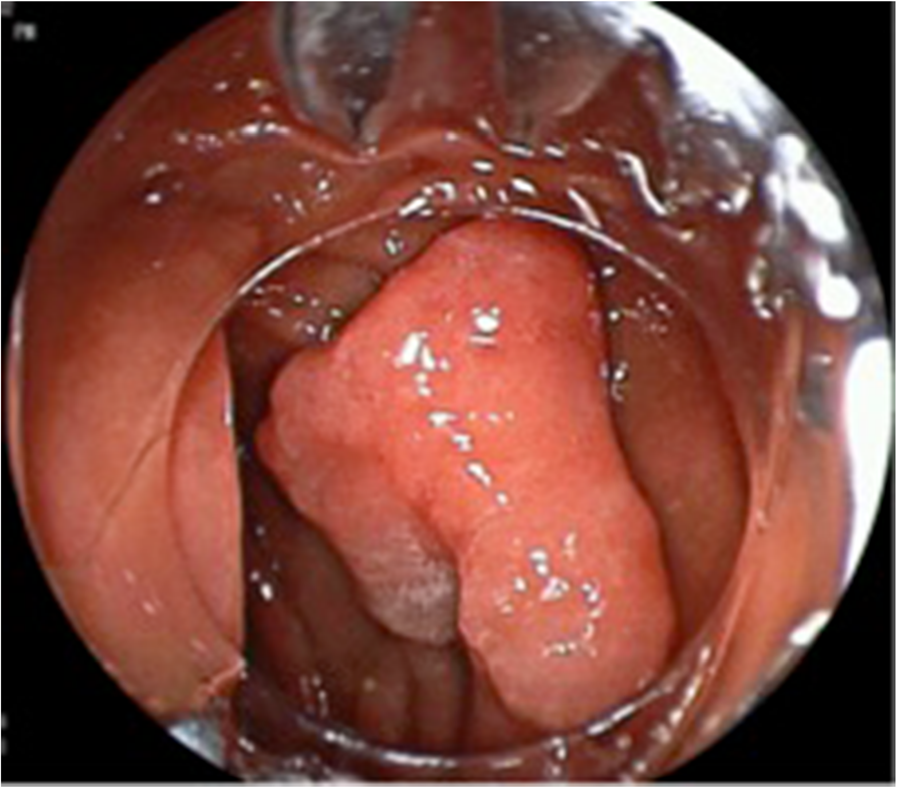
Jejunal polyp seen at DBE

**Fig. 2:**
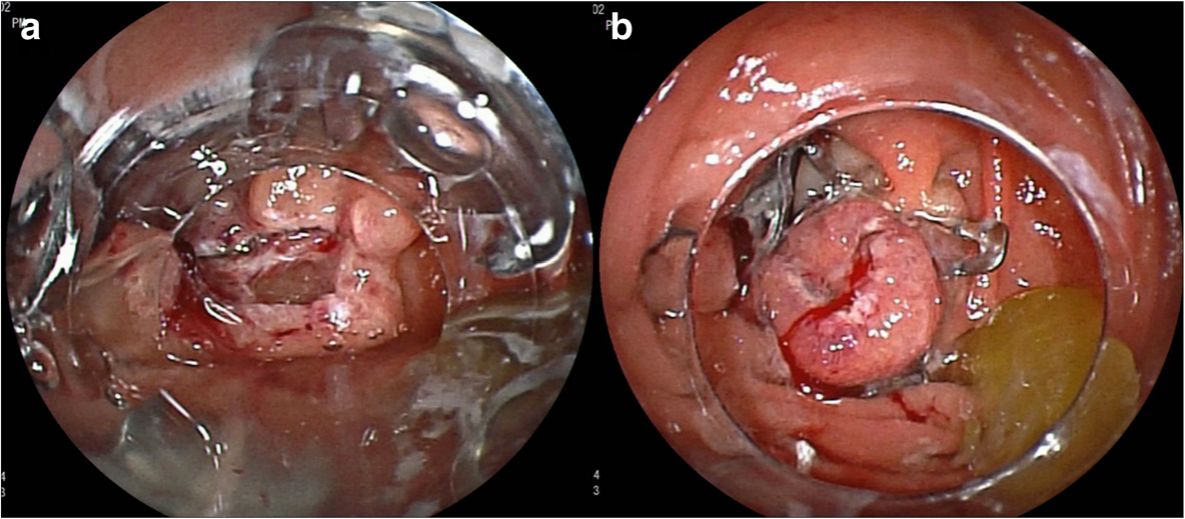
**a**: Jejunal full thickness defect after EMR polypectomy; **b**: Jejunal perforation closure with OTSC system

The cap of OTSC was mounted onto the distal tip of a DB enteroscope (EI-530B, Fuji, Tokyo, Japan). The DB enteroscope was introduced through the mouth and advanced down to proximal jejunum. The flat-elevated polyp was identified (Fig. 1) and en-bloc EMR was performed using a bipolar snare (DRAGONARE, Zeon Medical, Tokyo, Japan), after the injection of EMR solution (glycerol, indigo carmine and epinephrine). As expected, the procedure was difficult, for the reasons stated above. Once the polyp was resected, a full-thickness defect of about 5 mm (Fig. 2a) was visible on the base of EMR polypectomy. After careful retraction of the edges into the cap, the OTSC device was directed towards the defect, the clip was deployed and the perforation was successfully closed (Fig. 2b). The resected polyp was grabbed with grasping forceps and pulled into the plastic cap for retrieval; in this way, the specimen was not damaged. The histology did not show any evidence of malignancy.

**Fig. 3:**
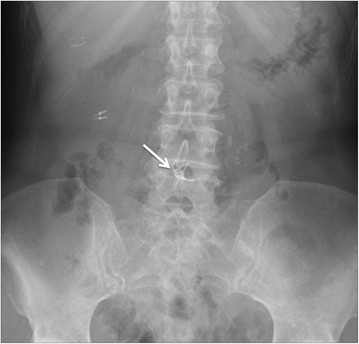
Plain X ray of the abdomen two years after the procedure, showing OTSC still in place (see arrow)

We monitored patient’s clinical condition after the procedure. Routine blood test and a plain X-ray of the abdomen did not show any abnormalities. She did not experience any GI symptoms; the blood pressure and temperature were within normal values. We only administered intravenous fluids; no antibiotics were used. The day after the procedure she was allowed to drink water and, since she remained asymptomatic, she was allowed a liquid diet two days after EMR polypectomy. She was discharged home four days after the procedure, able to tolerate a normal diet and with no complication. Two years later, patient underwent surveillance CE, which did not show any recurrence, and a plain X-ray of the abdomen, which showed the OTSC still in place (Fig. 3).

## Discussion and conclusions

FAP is a rare inherited disease, clinically characterized by numerous adenomatous polyps throughout the colon. Prophylactic colectomy has decreased the incidence of colorectal cancer in FAP patients, and duodenal cancer is now the leading cause of death [7]. Adenomas beyond duodenum may occur throughout the small bowel, but are mostly located in the proximal jejunum and distal ileum [3].

Endoscopic treatment of jejunal polyps can be tricky and is associated to a high risk of perforation. Indeed, jejunum has a thinner wall, compared to the rest of GI tract, and is located in a difficult anatomical position, which makes the stability and control of the scope complicated, especially during therapeutic procedures, such as EMR. In our patient, the risk of perforation was even higher as, in addition to the reasons explained above, she previously underwent abdominal surgery (IRA and IPAA), with consequent adhesions formation, and a further technical difficulty in performing therapeutic endoscopy. Moreover, the jejunal polyp had a flat morphology, which further increased the possibility of perforation. In light of such considerations, we had to carefully think in advance about a feasible endoscopic treatment, which would ensure a rapid and safe management in case of jejunal perforation following EMR. The placement of traditional through-the-scope clips in a narrow space like jejunum is usually technically difficult and time consuming. Recent studies showed successful GI perforation closure with OTSC system in clinical cases [8, 9] and only one case report in literature showed the efficacy of OTSC in treating a perforation specifically located in jejunum [10]. In our case, we prepared in advance the OTSC system, which was mounted onto the distal end of a DB enteroscope before starting the procedure. By doing so, when the jejunal perforation occurred after EMR, we had the OTSC ready to be placed. In this way, we could act fast and an enlargement of the defect was avoided. The OTSC was easily deployed and perforation was completely closed. Patient remained asymptomatic after the procedure and was able to rapidly resume oral intake and go back home with no complication.

In light of such results, we would say that the OTSC system, pre-mounted onto a DB enteroscope, was a safe alternative to conventional metal clips in our patient. Indeed, although the jejunum is usually a tricky place to perform therapeutic endoscopy, the OTSC system allowed a fast management of jejunal perforation, by enabling a complete closure of the defect in a relatively easy way.

## Abbreviations

APC: Adenomatous polyposis coli
CE: Capsule endoscopy
DB: Double balloon
EGD: Esophagogastroduodenoscopy
EMR: Endoscopic mucosal resection
FAP: Familial adenomatous polyposis
GI: Gastrointestinal
IPAA: Ileal pouch-anal anastomosis
IRA: Ileorectal anastomosis
OTSC: Over-The-Scope-Clip

## References

[1] Syngal S, Brand RE, Church JM, Giardiello FM, Hampel HL, Burt RW, American College of Gastroenterology (2015). ACG clinical guideline: genetic testing and management of hereditary gastrointestinal cancer syndromes. Am J Gastroenterol.

[2] Vasen HF, Möslein G, Alonso A, Aretz S, Bernstein I, Bertario L (2008). Guidelines for the clinical management of familial adenomatous polyposis (FAP). Gut.

[3] Alderlieste YA, Rauws EA, Mathus-Vliegen EM, Fockens P, Dekker E (2013). Prospective enteroscopic evaluation of jejunal polyposis in patients with familial adenomatous polyposis and advanced duodenal polyposis. Familial Cancer.

[4] Burke CA, Santisi J, Church J, Levinthal G (2005). The utility of capsule endoscopy small bowel surveillance in patients with polyposis. Am J Gastroenterol.

[5] Mönkemüller K, Fry LC, Ebert M, Bellutti M, Venerito M, Knippig C (2007). Feasibility of double-balloon enteroscopy-assisted chromoendoscopy of the small bowel in patients with familial adenomatous polyposis. Endoscopy.

[6] Groves CJ, Saunders BP, Spigelman AD, Phillips RK (2002). Duodenal cancer in patients with familial adenomatous polyposis (FAP): results of a 10 year prospective study. Gut.

[7] Latchford AR, Neale KF, Spigelman AD, Phillips RK, Clark SK (2009). Features of duodenal cancer in patients with familial adenomatous polyposis. Clin Gastroenterol Hepatol.

[8] Matthes K, Jung Y, Kato M, Gromski MA, Chuttani R (2011). Efficacy of full-thickness GI perforation closure with a novel over-the-scope clip application device: an animal study. Gastrointest Endosc.

[9] Parodi A, Repici A, Pedroni A, Blanchi S, Conio M (2010). Endoscopic management of GI perforations with a new over-the-scope clip device (with videos). Gastrointest Endosc.

[10] Samarasena J, Chen CL, Chin M, Chang K, Lee J (2017). Successful closure of a cryotherapy-induced bleeding jejunal perforation with the over-the-scope clip system. Gastrointest Endosc.

